# Iridescence Mimicking in Fabrics: A Ultraviolet/Visible Spectroscopy Study

**DOI:** 10.3390/biomimetics9020071

**Published:** 2024-01-25

**Authors:** Rui D. V. Fernandes, Alina Pranovich, Sergiy Valyukh, Andrea Zille, Tomas Hallberg, Kenneth Järrendahl

**Affiliations:** 1 Centre for Textile Science and Technology (2C2T), University of Minho, 4800-058 Guimarães, Portugal; rdvfernandes@2c2t.uminho.pt; 2 Department of Science and Technology (ITN), Linköping University, SE-601 74 Norrköping, Sweden; alina.pranovich@liu.se; 3 Media and Information Technology (MIT), Linköping University, SE-601 74 Norrköping, Sweden; 4 Department of Physics, Chemistry and Biology (IFM), Linköping University, SE-581 83 Linköping, Sweden; sergiy.valyukh@liu.se; 5 Division of Electromagnetic Warfare, Swedish Defense Research Agency (FOI), SE-583 30 Linköping, Sweden; tomas.hallberg@foi.se

**Keywords:** photonic crystals, structural coloration, iridescent effect, textiles, UV/Vis reflectance, IP-BRDF

## Abstract

Poly(styrene-methyl methacrylate-acrylic acid) photonic crystals (PCs), with five different sizes (170, 190, 210, 230 and 250 nm), were applied onto three plain fabrics, namely polyamide, polyester and cotton. The PC-coated fabrics were analyzed using scanning electronic microscopy and two UV/Vis reflectance spectrophotometric techniques (integrating sphere and scatterometry) to evaluate the PCs’ self-assembly along with the obtained spectral and colors characteristics. Results showed that surface roughness of the fabrics had a major influence on the color produced by PCs. Polyamide-coated fabrics were the only samples having an iridescent effect, producing more vivid and brilliant colors than polyester and cotton samples. It was observed that as the angle of incident light increases, a hypsochromic shift in the reflection peak occurs along with the formation of new reflection peaks. Furthermore, color behavior simulations were performed with an illuminant A light source on polyamide samples. The illuminant A simulation showed greener and yellower structural colors than those illuminated with D50. The polyester and cotton samples were analyzed using scatterometry to check for iridescence, which was unseen upon ocular inspection and then proven to be present in these samples. This work allowed a better comprehension of how structural colors and their iridescence are affected by the textile substrate morphology and fiber type.

## 1. Introduction

Human beings have been trying to imitate Nature characteristics in laboratory since the early stages of modern science. Some optical characteristics can be achieved through structural coloration and photonic crystals (PCs). PCs are innovative materials inspired by nature, particularly the iridescent colors of butterfly bird feathers, and beetle exoskeletons having color generating structures in one, two, or three dimensions [1,2,3]. These artificial structures mimic the intricate patterns found in biological organisms to manipulate and control light. By emulating nature’s designs, photonic crystals exhibit unique optical properties that can bend, reflect, or trap light of specific wavelengths.

PCs are periodic structures that exhibit a bandgap for certain wavelengths of the electromagnetic radiation, similar to how semiconductors have electronic bandgaps for electron energy levels [4,5]. The photonic bandgap (PBG) arises from the periodic variation in refractive index within the structure, where light is forbidden to propagate within the PCs, thus being determined by the crystal lattice and periodicity. Depending on material choices for the PCs synthesis (polystyrene (PS) or silica (SiO_2_) particles) and the substrate (glass, metallic, or fabric), structural colors with iridescent effects can occur due to angular resolved Bragg diffraction from the lattice planes [6]. PCs can be applied in ink-jet printing [7,8], 3D printing [9,10], lithography [11,12] and sensors [13,14], among others [15,16], to produce structural colors. They can also be applied in textiles, an area of research that had demonstrated great interest in the last decade [6,17,18,19,20]. Being one of the most pollutant industries in the world, there is a constant demand that the textile industry searches for more ecological methods of coloration, using less chemicals and water. One promising approach to a more ecological production of colored fabrics is the application of structurally coloring PCs where, compared to traditional dyeing processes, the consumption of water is dramatically reduced. The reason is that there is no need for water to rinse the fabrics after coloring, thus effluents can be severely reduced. However, a fabric surface is very irregular when compared to glass or metallic surfaces, so applying PCs on fabrics can be challenging since, the fiber (natural or synthetic, shape, cross-section area), yarn properties (number of cables, torsion, linear density) and fabric construction (number of yarns at warp and weft, type of structure) will influence its surface roughness. Li et al. [21] studied the influence of weave, yarn diameter and yarn density of polyester and silk fabrics on obtaining SiO_2_-based structural colors. It was observed that both physical and chemical properties of the fabric influence structural color. Li et al. observed that, physically, more compact and flat substrates would promote better PC self-assembly, meaning that plain fabrics were better substrates than twill fabrics due to the small gaps between fibers, for both polyester and silk. Chemically, fabrics that possess less polar and water-soluble groups in their composition will be preferred to achieve better structural colors, which in this study were exemplified with polyester fabrics. Zhou et al. [21] also performed structural coloration studies on polyester fabrics, with variations on the PC’s mass fraction utilized for the coating, humidity percentage and temperature [22]. It was noted that using a mass fraction of 1.5% of SiO_2_ PCs led to better arranged structures versus mass fractions of 0.5, 1.0 and 2.5%. Furthermore, higher relative humidity percentages (60%) and lower temperatures (25 °C) also led to better organized structures meaning the evaporation rate of the PCs colloidal solution, liquid retention and stability of the fabrics (shrinkage) are determinant factors to obtain good structural colors.

In this work, we highlight the importance of truly understanding the color behavior of PCs structures, especially when applied in different types of fabrics with different surfaces roughness. In this context, we investigate 3 plain fabrics of different fibers (cotton, polyamide, polyester) structurally colored with five different sizes of poly(styrene-methylmethacrylate-acrilic acid) (P(St-MMA-AA)) PCs (170, 190, 210, 230 and 250 nm). In all cases we evaluated the final structural color and how the coloring was influenced by the properties of the fabric. To the best of our knowledge, there are very few research papers that use polyamide fabrics in structural coloration studies, and even less studies comparing the differences of (P(St-MMA-AA)) PCs applied onto polyamide, polyester and cotton fabrics. Furthermore, there are no studies about structural color behavior in textiles under other illuminants, hence the importance of the performed research. The future of textile coloring will certainly depend on photonic crystals and their structural color, so that we have a color that, in addition to being more ecological and sustainable, is also capable of mimicking optical effects produced by nature.

## 2. Materials and Methods

### 2.1. Materials

Commercial black polyamide (PA) plain fabric (61.50 g/m^2^), white polyester (PES) plain fabric (Lemar–Leandro Magalhães de Araújo (Filhos), Lda, Guimarães, Portugal, 100 g/m^2^) and white cotton (CO) plain fabric (Lameirinho Indústria Têxtil SA, Guimarães, Portugal, 140 g/m^2^) were used for dyeing processes and PC coating. Polyester and cotton dyeing (with black dye) were carried out in an Ibelus C-720 (Pregitzer&Ca, LDA, Guimarães, Portugal) dyeing machine equipped with infrared heating. P(St-MMA-AA) microspheres were previously synthetized accordingly [18] and used for the PC coating without any purification.

### 2.2. Fabrics Characterization

Polyamide, polyester and cotton fabrics were characterized according to the International Organization of Standardization (ISO) as follows (n = 3).

- ISO 7211-2:1984–Textiles–Woven fabrics–Construction–Methods of analysis–Part 2: Determination of number of threads per unit length [23].
- ISO 7211-3:1984–Textiles–Woven fabrics–Construction–Methods of analysis–Part 3: Determination of crimp of yarn in fabric [24].
- ISO 7211-5:2020–Textiles–Methods for analysis of woven fabrics construction–Part 5: Determination of linear density of yarn removed from fabric [25].

Thickness measurements were performed with an electronic digital micrometer (Mitutoyo) in 5 different points of the fabric, for all fabrics (n = 3).

### 2.3. Cotton Dyeing

**Figure 1:**
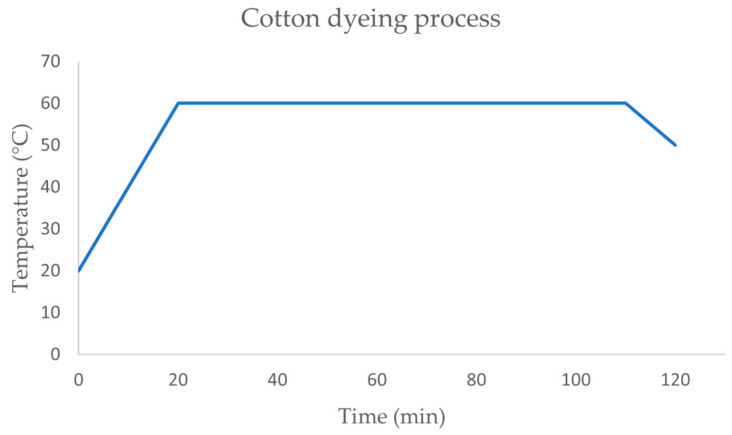
Dyeing process used for CO at 60 °C.

Cotton plain fabric was dyed with Novacron Black NS (Huntsman Performance, Leça da Palmeira, Portugal) at 6% over fiber weight (ofw). The dyeing bath was composed by 100 g/L of NaCl, 5 g/L of Na_2_CO_3_, 3 mL/L of NaOH 50%, with a liquor ratio of 1:20. The dyeing program (Figure 1) started at room temperature (~20 °C) and then was raised to 60 °C, which was maintained for 90 min. After cooling to 50 °C, cotton fabric was rinsed in cold water to remove hydrolyzed dye from the fabric’s surface, washed at 100 °C for 20 min with detergent Diadavin UN (Tanatex Chemicals, Santo Tirso, Portugal) 1 g/L to remove the remaining hydrolyzed dye in the fabric’s interior, rinsed again with cold water and then dried in an oven at 40 °C.

### 2.4. Polyester Dyeing

**Figure 2:**
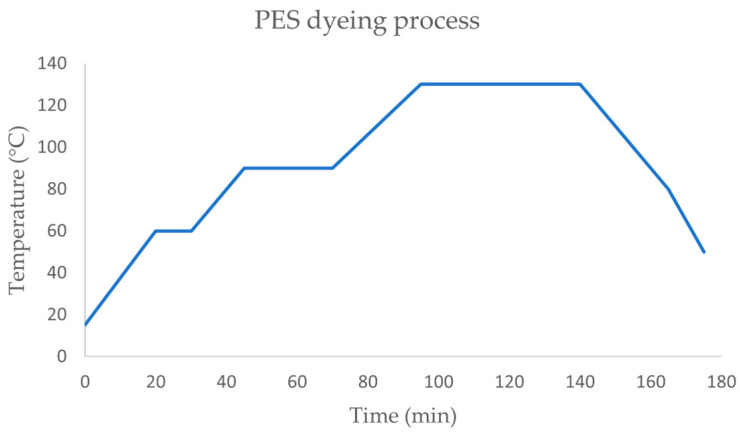
Dyeing process used for PES at 130 °C.

Polyester plain fabric was dyed with Coralene Black MD (IMPOCOLOR, Grijó, Portugal) at 3% ofw, with a liquor ratio of 1:30. The dyeing process (Figure 2) started at room temperature (~20 °C), with a subsequent raise of the temperature in several steps: to 60 °C with a rate of 3 °C/min and kept at 60 °C for 10 min; to 90 °C with a rate of 2 °C/min and kept at 90 °C for 30 min; to 130 °C with a rate of 1.5 °C/min and kept at 130°C for 45 min. The cooling was performed in two steps, from 130 °C to 80 °C with a rate of 2 °C/min and then from 80 °C to 50 °C with a rate of 2.5 °C/min. Finally, the PES fabrics were rinsed with hot water followed by rinsing with cold water and dried in an oven at 40 °C.

### 2.5. PCs Deposition by Dip-Drawing Method

A piece of fabric, 3 × 3 cm^2^, was dipped in the P(St-MMA-AA) disperse solution (15% wt) for 10 s, slowly removed and dried in a petri dish at 40° C for 15–25 min. Fabrics were used without any further modifications for UV/Vis reflectance measurements.

### 2.6. Reflectance Measurements

#### 2.6.1. Spectrophotometry with Integrating Sphere

The PC coated fabrics were analyzed with a spectrophotometer (Cary 5000, Agilent, Santa Clara, CA, USA) equipped with an integrating sphere (DRA 2500, Agilent, Santa Clara, CA, USA), within the wavelength range from 250 to 2500 nm (NUV-VIS-NIR). However, only the wavelength region 350–750 nm (VIS) was analyzed in this work. Directional Hemispheric Reflectance (DHR) was both measured at a fixed angle of incidence of 8 degrees (total DHR, without polarizer) and at different angles of incidence ($\alpha_{i}$), from 12 to 80 degrees, for both s- and p-polarized light.

#### 2.6.2. Scatterometry

**Figure 3:**
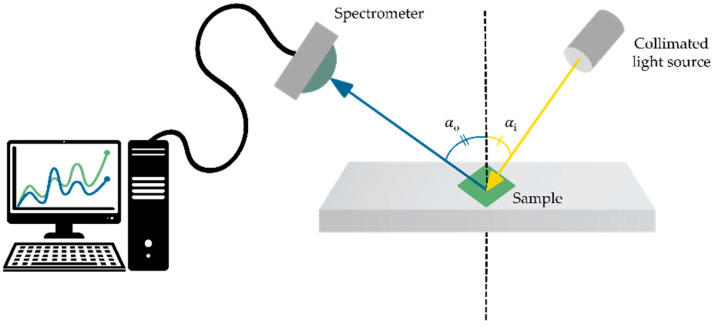
Schematic of IP-BRDF setup.

For color characterization of the fabric samples, scatterometry measurements were first performed giving the In-Plane Bidirectional Reflectance Distribution Function (IP-BRDF). These measurements were performed using a modified set up, based on an RC2 ellipsometer (J.A. Woollam Co., Inc., Lincoln, NE, USA), comprised of a spectrometer, light source, and goniometer. The measurements were made at incident angles $30{}^{\circ}\leq \alpha_{i}\leq 70{}^{\circ}$ in steps of 10°. For each incident angle the angle of observation was varied according to $0{}^{\circ}\leq \alpha_{o}\leq 70{}^{\circ}$ with varying steps. That is, each sample was measured with 20 settings of the incident and observation angles. Measured reflectance values were interpolated to cover the whole range of observation directions with 1° resolution. The light source used for these measurements was D50 illuminant. A schematic of the experimental setup with angle notation is shown in Figure 3.

### 2.7. Conversion of Data to 3D Color Coordinates and IP-BRDF Imaging

Since the experimental set up is using a spectrometer with unknown spectral sensitivity, a simple procedure of conversion to color coordinates was performed. The angularly varying spectral intensity of the white reference (99% Spectralon) was first recorded using the spectrometer. Then spectral reflectance values for the white reference were measured, and its reflectance was calculated as the ratio between two signals (Signal_white/Signal_lamp), with exposure time factor included. After collecting reflectance in arbitrary units for the measured samples, a normalization was made by dividing with the white reference signal (IP-BRDF_sample = Signal_sample/Signal_white). Finally, the obtained reflectance values were converted to the color coordinates under the assumption of illuminant (D50 or illuminant A), CIEL*a*b* or RGB color [26].

### 2.8. Scanning Electron Microscopy (SEM)

Morphological analyses of the PC-coated fabrics were carried out by scanning electron microscopy with an ultra-high resolution field emission gun (FEI NOVA 200 Nano SEM). Before all measurements, the PC-coated fabric samples were covered with a thin film of gold/palladium (80:20) in a 208HR high-resolution sputter coater (Cressington, Watford, UK) coupled to an MTM-20 high-resolution thickness controller (Cressington, Watford, UK).

### 2.9. Surface Roughness

Surface roughness was measured with a HANDYSURF^+^ 35/40/45 (ACCRETECH) rugosimeter. Calibrations parameters and calculations were defined according to standard ISO 21920-2:2021 [27], using a Gaussian filter. Roughness average (Ra) measurements were performed at a speed of 0.6 mm/s for a length of 10 mm. Each Ra value is an average of 12 points measured along the 10 mm of fabric. Measurements were performed in triplicate for each sample.

### 2.10. Photographs

Digital photographs of uncoated and coated fabrics with PCs were taken with a Canon EOS M50 (Canon Portugal S.A, Porto Salvo, Portugal) digital camera. The images were acquired in a light chamber under a D65 light source, maintaining the same distance for all samples. No adjustments of pixels, color, brightness or contrast were applied to the images. The photographs produced were parts of larger images that were selected without obscuring, eliminating or misrepresenting any information that was present in the originals.

## 3. Results and Discussion

To understand if any type of textile substrate is suitable (without any pretreatment) to mimic the structural colors found in nature, all three fabrics were characterized, in terms of thickness, linear density of the yarns (measured in Tex, where 1 Tex = 1 g per 1 km) at warp and weft, and number of yarns per centimeter at warp and weft (Table 1), before any procedure was performed on the fabrics.

**Figure 4:**
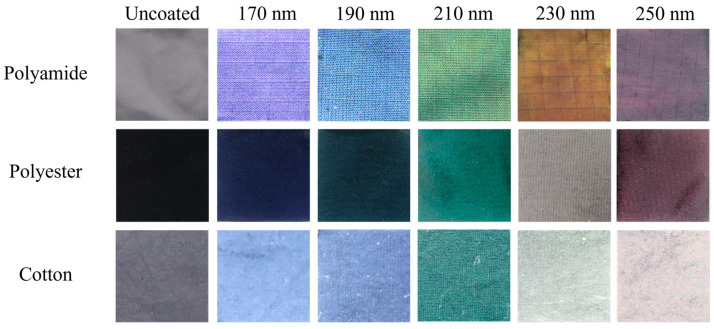
Digital photographs of uncoated polyamide (PA, L* = 21.11, a* = 0.52, b* = −1.13), polyester (PES, L* = 20.36, a* = −0.34, b* = −3.13) and cotton (CO, L* = 21.19, a* = 0.80, b* = −3.97) fabrics, and PA, PES and CO fabrics coated with 170, 190, 210, 230 and 250 nm PCs. Note: the uncoated PA and CO appear grey in photographs but are black.

To enhance the chroma (color intensity, saturation, or purity) from the structural colors, cotton and polyester fabrics were dyed black, as described above, whereas black polyamide fabric was commercially available [6,17,21,28,29]. Then, P(St-MMA-AA) photonic crystals with five different sizes (170, 190, 210, 230 and 250 nm) were applied by dip coating onto polyamide, polyester, and cotton fabrics, to produce violet, blue, green, green-red and red structural colors (Figure 4), respectively.

**Figure 5:**
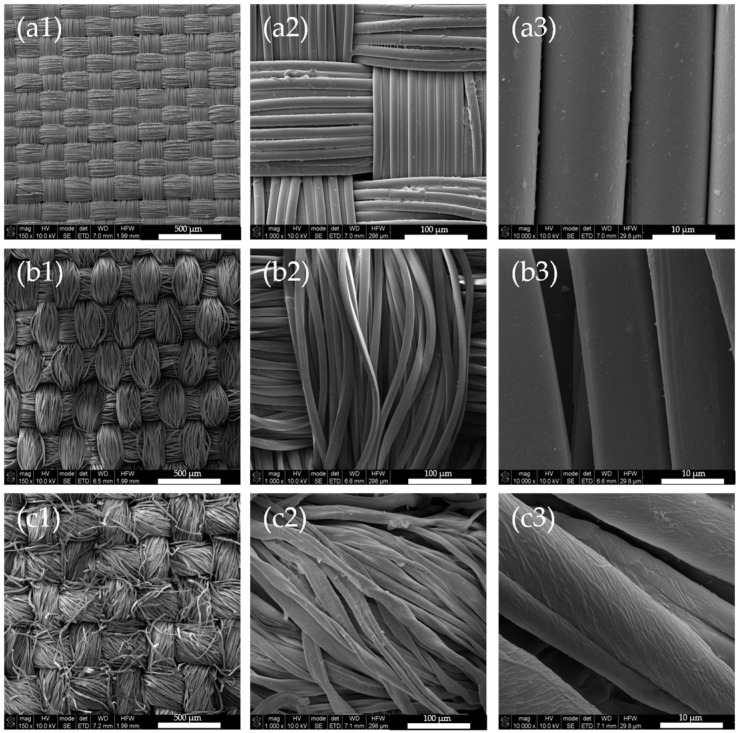
SEM micrographs of polyamide (row **a**), polyester (row **b**) and cotton (row **c**) uncoated fabrics at 150× (**a1**,**b1**,**c1**), 1000× (**a2**,**b2**,**c2**) and 10,000× (**a3**,**b3**,**c3**) magnifications.

**Figure 6:**
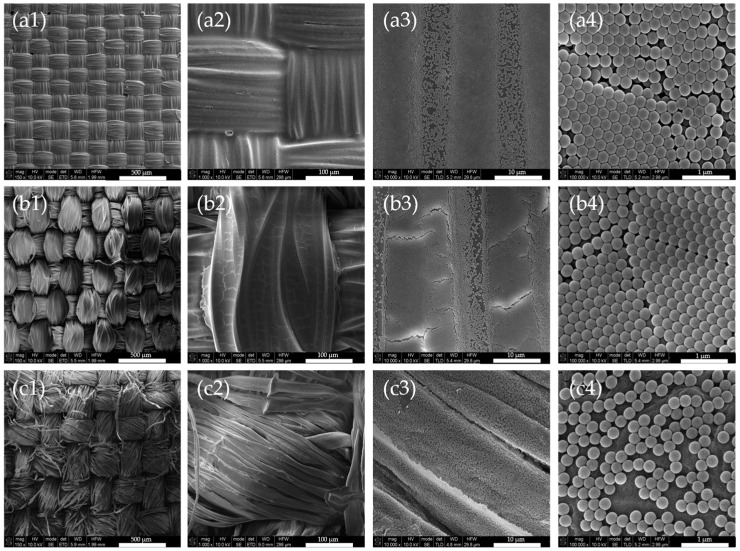
SEM micrographs of polyamide (row **a**), polyester (row **b**) and cotton (row **c**) coated fabrics with 190 nm PCs at 150× (**a1**,**b1**,**c1**), 1000× (**a2**,**b2**,**c2**), 10,000× (**a3**,**b3**,**c3**) and 100,000× (**a4**,**b4**,**c4**) magnifications.

As seen in Figure 4, the polyamide samples exhibit brighter colors than polyester and cotton samples. Upon ocular inspection of all three fabric samples, PA samples are the only ones to have a visible iridescent effect. Furthermore, cotton samples exhibit a milky aspect at the fabric’s surface. Since all three fabrics have the same plain structure, differences in the sample’s chromaticity are probably due to the fiber’s type and linear density (g/m^2^). As both polyamide and polyester are synthetic fibers, the difference in color must be related to the linear density of the fabrics and consequently, surface roughness. Fabrics composed of natural fibers, such as cotton, usually have a rougher surface than synthetic ones. To assess these assumptions, SEM and surface roughness measurements were performed on all three fabrics, for both coated and uncoated samples. Figure 5 shows SEM micrographs of the fabrics utilized for PC deposition at different magnifications, whereas Figure 6 denotes the same fabrics coated with 190 nm PCs.

When analyzing the SEM micrographs, uncoated fabrics seems to show an increase of surface roughness, with polyamide being the smoothest, followed by polyester and cotton. To confirm these visual assumptions, the surface roughness average (Ra) of uncoated fabrics was measured with a HANDYSURF^+^ rugosimeter. The results are presented in Table 2, along with the surface roughness of PC coated fabrics. Surface roughness values are in agreement with SEM micrographs, where polyamide is in fact the smoothest fabric with an Ra of 6.33 ± 1.99 µm, followed by polyester with an Ra of 16.43 ± 5.51 µm and cotton, the roughest fabric with an Ra of 17.43 ± 6.27 µm. Polyamide SEM analysis also showed that, the levelling agent used in the dyeing process may created a coating, but due to its dimension, was assumed to not affect surface roughness, taking in account the relative dimension of the PCs. After PC coating, polyamide and cotton fabrics maintained their Ra values. Polyester Ra increased by 3 µm after the PC coating, which is less than one third of the diameter of one individual thread (~10 µm) of the fabric’s yarn (~250 µm warp, ~200 µm weft), thus this increase may be disregarded.

Since SEM micrographs of PES samples showed a very well-organized structure, it was expected that these samples should present bright colors and iridescence. However this was not the case, probably due to the roughness factor. Instead, the PA samples were the brightest, with the most intense color and the only ones to present visible iridescence from ocular inspection. Since all three fabrics have the same plain structure, the factors that are influencing structural coloration are the thickness, yarn Tex and the tightness of the weave. The combination of all these factors will affect the fabric surface roughness, which should be as low as possible for a good and uniform photonic crystal deposition. Thus, after analyzing these fabric properties it is concluded that a lower fabric thickness, low yarn Tex and a tight weave will produce fabrics with lower surface roughness. If fabric thickness is low, there will be less PC spheres needed to fill gaps between fibers, thus more spheres are available to form a more uniform and flat coatings. Yarns with low Tex values are thinner, smoother and with less fibrils on its surface, thus roughness can be minimized, to an extent, without compromising its physical and mechanical properties. Furthermore, in tighter weaves the gaps between the fibers are smaller, leading to a higher fabric cover, which will diminish surface roughness and increase the surface area for a better PC deposition, without defaults/cracks in its structure. These conclusions are in agreement with the studied performed by Li et al., where polyester and silk substrates with different structures, plain and twill, were analyzed for PC deposition purposes and, consequently, optimization of structural color [21]. It was observed that plain structures were preferred over twill structures, for both polyester and silk, due to the higher covering factor of this type of structure. Plain structures with tighter weaves lead to fabrics that are more compact, flat, and dimensionally stable, which demonstrated to be better for the obtention of good structural colors. Furthermore, yarn diameter and yarn density were determinant factors to obtain the best structural color possible. Thus, it is of high importance to know what are the characteristics of the textile substrate used for PC biomimicry [30,31].

**Figure 7:**
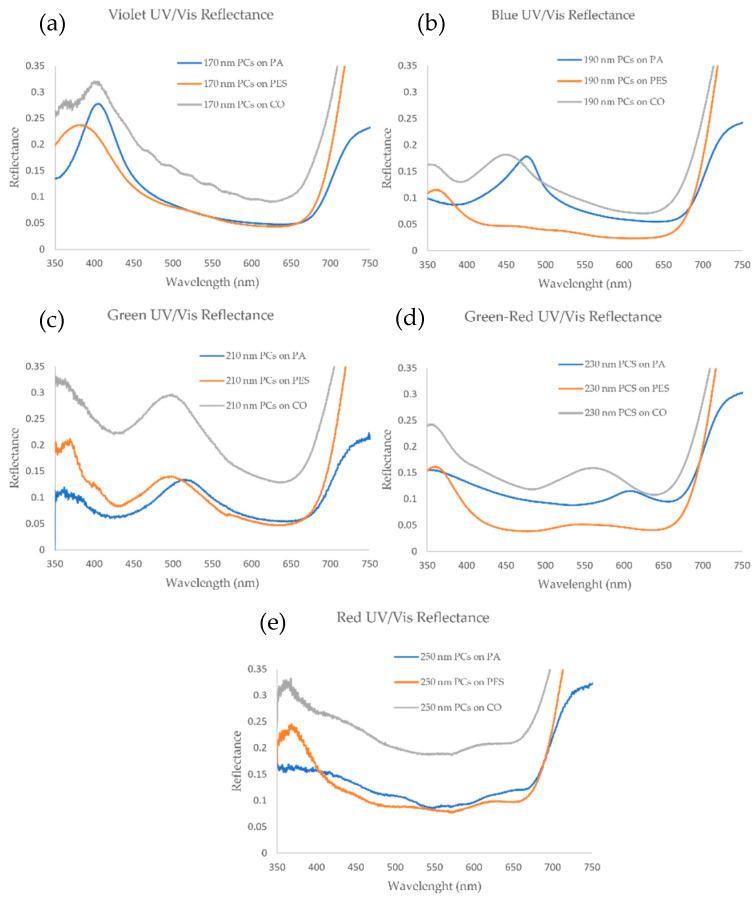
Fixed angle of incidence (8°) UV/Vis reflectance (DHR) spectra for (**a**) 170, (**b**) 190, (**c**) 210, (**d**) 230 and (**e**) 250 nm PCs in polyamide, polyester and cotton fabrics.

After comprehending photonic physical behavior in different types of fibers, the PC-coated polyamide, polyester and cotton fabrics were analyzed by UV/Vis reflectance measurements for each color/particle size (Figure 7).

The PA samples produced sharp reflection peaks in the visible wavelength region, except for the red sample, where the peak is very weak for all three fabrics. In most cases, the CO samples have higher reflectance as compared to PA and PES since the self-assemble structures in these samples are not uniform, giving them a whiter appearance, due to higher light scattering on the fabric surface. Also, in CO samples, the highest reflection peak has a small hypsochromic (blue) shift when compared to PA samples. For PES samples, intensity reflectance is comparable to violet, green and red PA samples, always with a slight blue shift in terms of maximum reflection peak, as in CO samples.

Interestingly, in the fabrics coated with 230 nm PCs (green-red sample), the reflection peak appears in the green region for CO and PES, while in PA the peak appears in the red region. Thus, it can be assumed that, since SEM and roughness analysis showed that PA is the best substrate of all three fabrics for PC deposition, the main reflected color produced by the photonic structure is red. This could be used as a preliminary indication whether the self-assembly of PCs is good or not. By observing when the reflected color does not match between fabrics with PCs of the same size, the use of more expensive techniques such as SEM can be reduced.

**Figure 8:**
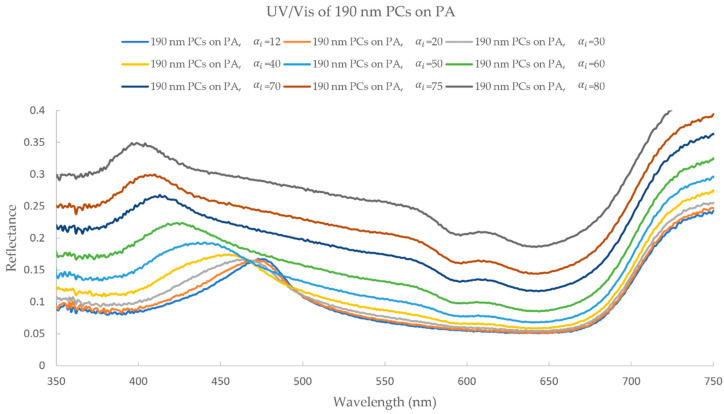
UV/Vis reflectance (s-polarized light) spectra analysis of PA coated with 190 nm PCs, with $\alpha_{i}$ ranging from 12° to 80°.

Since only the coated PA fabrics presented iridescence (by ocular inspection), angle-dependent UV/Vis reflectance measurements were done on only these samples. The measurements were performed for all five different colors, with both s- and p-polarized light. With this analysis it was possible to observe the reflection peak movement while changing the angle of incidence of light. PA samples coated with 190 (blue) and 230 nm (green-red) PCs presented the most interesting results, which will be discussed in more detail below. For PA coated with 190 nm PCs, for s-polarized light measurements, the initial reflection peak ($\alpha_{i}$ = 12°) appears at a wavelength of 475 nm (blue region), which moves with a hypsochromic shift as the $\alpha_{i}$ increases. At $\alpha_{i}$ = 80°, the reflection peak is located at a wavelength of 400 nm (violet region) (Figure 8). This behavior occurs similarly for p-polarized light, with the exception that above an $\alpha_{i}$ of 70° the reflection peak is no longer visible. Furthermore, in s-polarized light, a second reflection peak starts to appear at 610 nm (red region), from $\alpha_{i}$ of 50° to 80°, indicating that, at higher $\alpha_{i}$, the final color observed is the result of the addition of two separate colors (violet and red). This phenomenon is not observed in the p-polarized light measurements.

**Figure 9:**
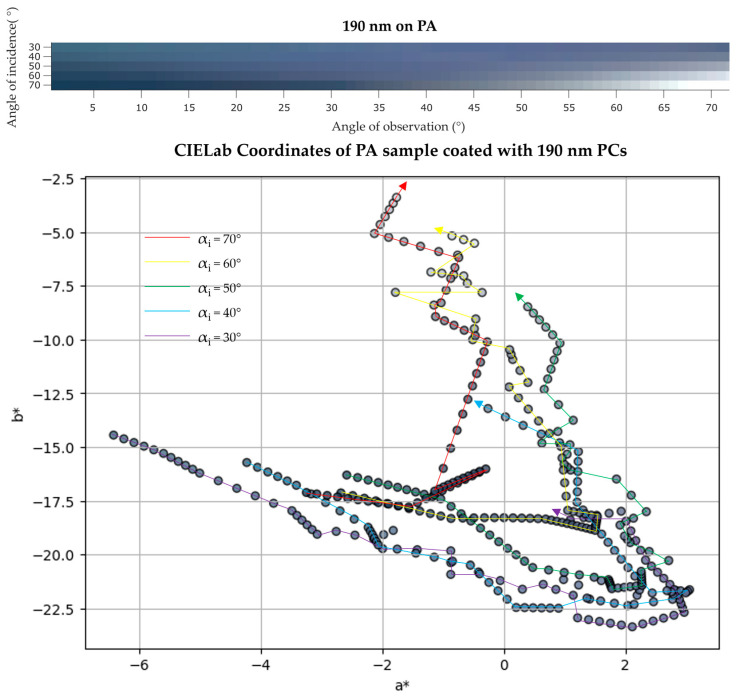
(**top**) IP-BRDF measurements of PA coated with 190 nm PCs, $\alpha_{i}$ = 30° to 70° and $\alpha_{o}$ = 0° to 70°; (**bottom**) 2D representation of color coordinates movement in CIEL*a*b* space, where the arrows represent the direction from low to high $\alpha_{o}$ (0° to 70°) for each $\alpha_{i}$ (30° to 70°).

In order to present the color appearance of the fabrics, scatterometry (or angular resolved reflectance spectroscopy) was recorded giving IP-BRDF data. With this technique it was possible to observe color shifts as the angle of incidence $\alpha_{i}$, and angle of observation $\alpha_{o}$ changes. As seen in Figure 9 (top), the initial color at an $\alpha_{i}$ = 30° is slightly green (at a low $\alpha_{o}$) and gradually changes to blue and violet as $\alpha_{i}$ and $\alpha_{o}$ increase. At a maximum $\alpha_{i}$ and $\alpha_{o}$, color sample is greyish, almost white, where the luminosity parameter L* increases drastically at these angles. This was observed for every sample tested. The IP-BRDF measurements are in alignment with the data observed in the UV/Vis spectra, thus validating both techniques for iridescence characterization. Color coordinates in a 2D CIEL*a*b* space are presented in Figure 9 (bottom), where the arrows represent the movement direction, from low to high $\alpha_{o}$. L* varied between 30 and 55, except at higher $\alpha_{i}$ and $\alpha_{o}$, which were above 80. This was also observed in the other PA samples.

**Figure 10:**
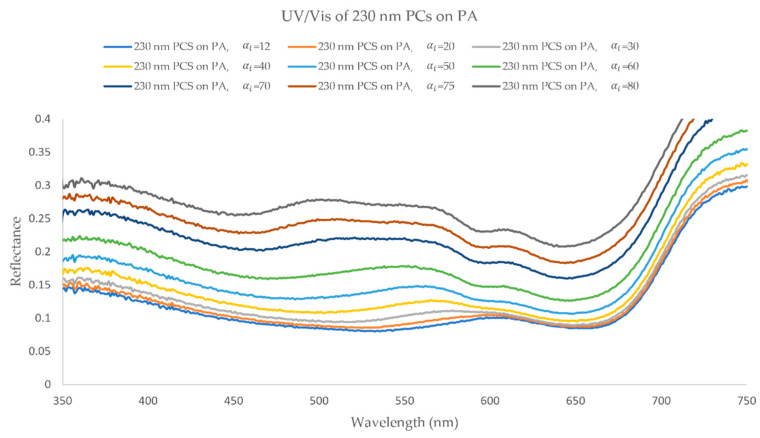
UV/Visible reflectance (s-polarized light) analysis of PA coated with 230 nm PCs, with $\alpha_{i}$ ranging from 12° to 80°.

The s-polarized light measurements of the PA sample coated with 230 nm PCs showed a single reflection peak for $\alpha_{i}$ = 12° at 610 nm (Figure 10). As the $\alpha_{i}$ increases, this peak stays unaltered at 610 nm and other reflection peaks start to appear. At $\alpha_{i}$ = 50° a new peak appears at 560 nm and, as the $\alpha_{i}$ increases to 80°, this peak is divided in two, where the first peak maintains its wavelength at 560 nm and a second one is formed at 500 nm. Similarly to the previous sample, this phenomenon is not noticeable in the p-polarized light measurements, where the peak at 610 nm fades away above $\alpha_{i}$ = 50° and the formation of new reflection peaks is not observed.

**Figure 11:**
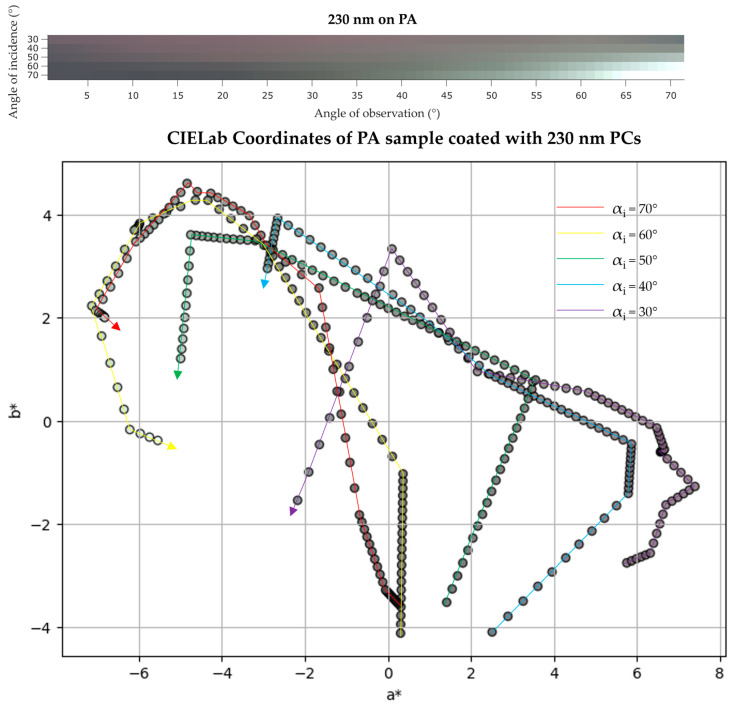
(**top**) IP-BRDF measurements of PA coated with 230 nm PCs, $\alpha_{i}$ = 30° to 70° and $\alpha_{o}$ = 0° to 70°; (**bottom**) 2D representation of color coordinates movement in CIEL*a*b* space, where the arrows represent the direction from low to high $\alpha_{o}$ (0° to 70°) for each $\alpha_{i}$ (30° to 70°).

The IP-BRDF measurements of the same sample (Figure 11 (top)), show that for low $\alpha_{o}$ the color is mainly red, for every $\alpha_{i}$. From $\alpha_{i}$ above 50°, at mid to high $\alpha_{o}$, the color changes to green. Once again, this is consistent with the angle-dependent UV/Vis data where a new peak appears at 560 nm (green region) at the same $\alpha_{i}$. Furthermore, this confirm the previous assumptions made in the fixed angle UV/Vis analysis, where the red color was dominant. As for CIEL*a*b* coordinates (Figure 11 (bottom)), L* varied between 38 and 58, and color is observed to be greener (lower a* values), yellower (higher b* values) at low $\alpha_{o}$ and bluer at high $\alpha_{o}$ (lower b* values).

**Figure 12:**
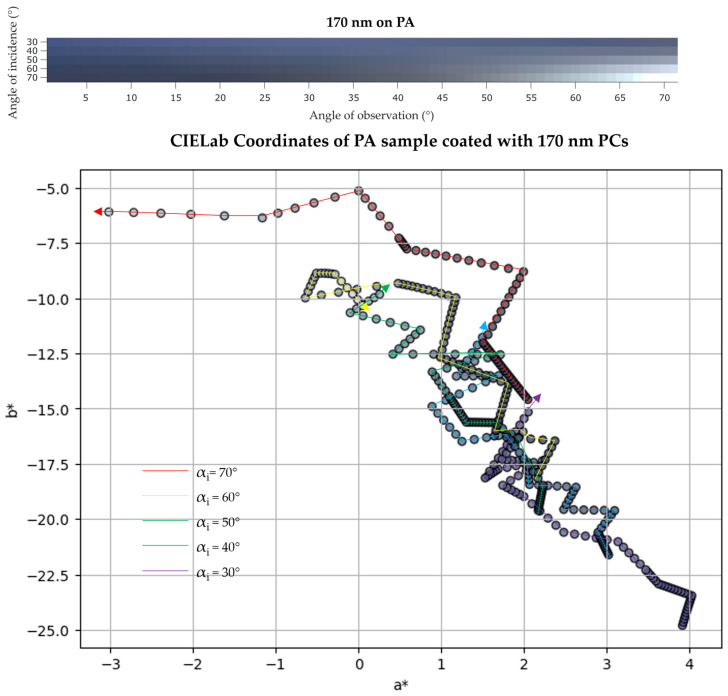
(**top**) IP-BRDF measurements of PA coated with 170 nm PCs, $\alpha_{i}$ = 30° to 70° and $\alpha_{o}$ = 0° to 70°; (**bottom**) 2D representation of color coordinates movement in CIEL*a*b* space, where the arrows represent the direction from low to high $\alpha_{o}$ (0° to 70°) for each $\alpha_{i}$ (30° to 70°).

**Figure 13:**
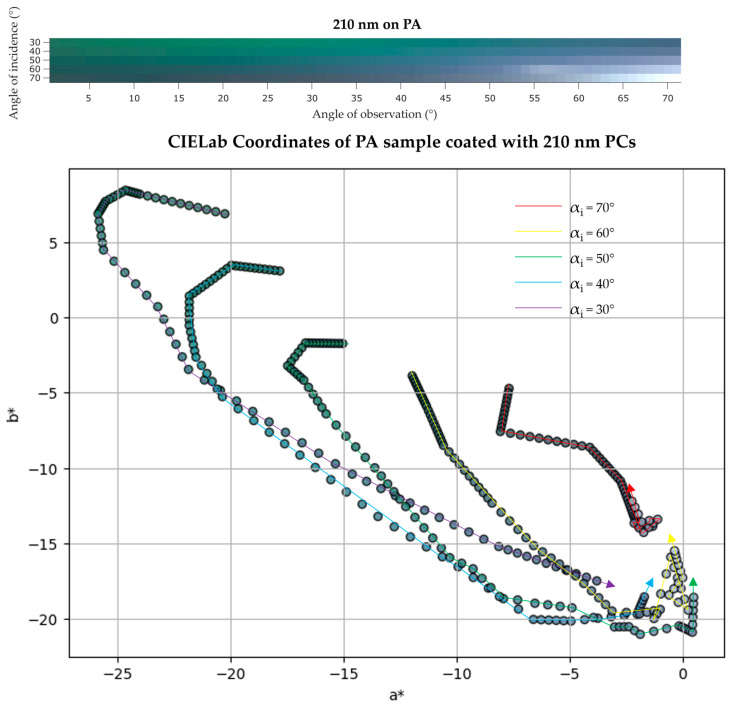
(**top**) IP-BRDF measurements of PA coated with 210 nm PCs, $\alpha_{i}$ = 30° to 70° and $\alpha_{o}$ = 0° to 70°; (**bottom**) 2D representation of color coordinates movement in CIEL*a*b* space, where the arrows represent the direction from low to high $\alpha_{o}$ (0° to 70°) for each $\alpha_{i}$ (30° to 70°).

**Figure 14:**
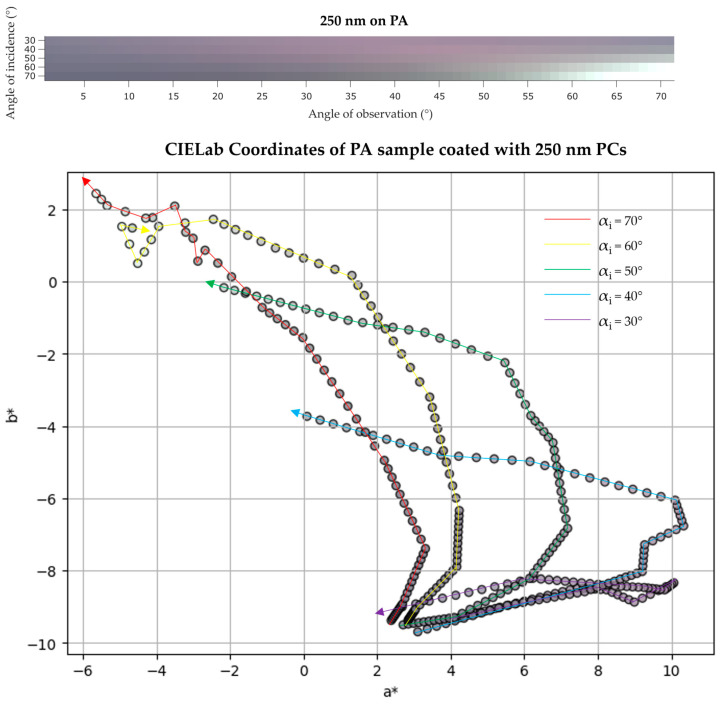
(**top**) IP-BRDF measurements of PA coated with 250 nm PCs, $\alpha_{i}$ = 30° to 70° and $\alpha_{o}$ = 0° to 70°; (**bottom**) 2D representation of color coordinates movement in CIEL*a*b* space, where the arrows represent the direction from low to high $\alpha_{o}$ (0° to 70°) for each $\alpha_{i}$ (30° to 70°).

UV/Vis reflectance measurements for PA samples coated with 170, 210, and 250 nm PCs were also performed. The UV/Vis data of these samples is briefly discussed along with IP-BRDF measurements and color coordinates analysis present below (Figure 12, Figure 13 and Figure 14). A summary of the UV/Vis peaks changes according to $\alpha_{i}$ is also presented in Table 3.

The PA sample coated with 170 nm PCs showed a small color change, the violet color (UV/Vis reflection data showed a peak at 405 nm for $\alpha_{i}$ = 20°) becomes lighter with higher $\alpha_{o}$ and darker at higher $\alpha_{i}$ (the peak at 405 nm shifted to 385 nm with increasing $\alpha_{i}$). Again, at a maximum $\alpha_{i}$ and $\alpha_{o}$ the sample color is basically grey, with L* parameter much higher than observed at other angles (L* ranged between 31 and 66). In the 2D color space, a yellower and greener color is obtained.

The PA sample coated with 210 nm PCs showed a color change from green (UV/Vis reflectance data showed a peak at 510 nm for $\alpha_{i}$ = 20°) to blue (the peak was slightly shifted to 490 nm for higher $\alpha_{i}$ and $\alpha_{o}$). This is consistent with the observed color coordinates, showing sample color getting bluer and redder as $\alpha_{i}$ increases. The luminosity varied between 34 and 63.

Finally, the PA sample coated with 250 nm PCs showed a color change from brown-red (UV/Vis reflectance data showed a peak at 640 nm for $\alpha_{i}$ = 20°) to green (a very weak peak appeared at 520 nm for $\alpha_{i}$ above 60° and the peak at 640 nm slightly shifted to 620 nm at higher $\alpha_{i}$). Analyzing the color space coordinates, it can be observed that at low $\alpha_{i}$ and $\alpha_{o}$, sample color is getting redder and slightly yellower. Then, at $\alpha_{i}$ above 50° and high $\alpha_{o}$, the sample color gets greener and yellower, which is in accordance with UV/Vis reflectance measurements. In terms of luminosity, this sample presented the highest values of all five samples, with L* values ranging between 48 and 80.

Overall, in all PA coated samples was observed a hypsochromic color shift as the $\alpha_{i}$ increased, in both UV/Vis and IP-BRDF analysis. PA samples showed a wide variety of colors, able to emulate the iridescent effects observed in nature.

**Figure 15:**
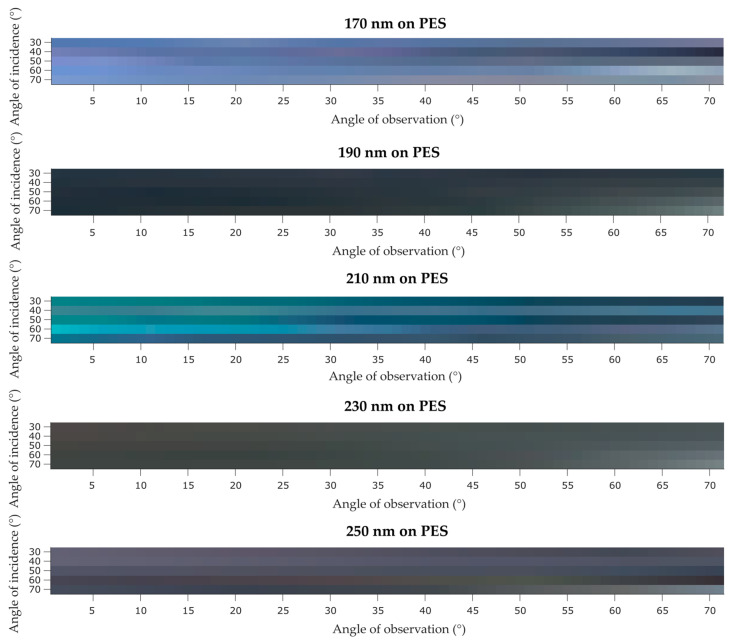
IP-BRDF measurements of PES coated samples.

**Figure 16:**
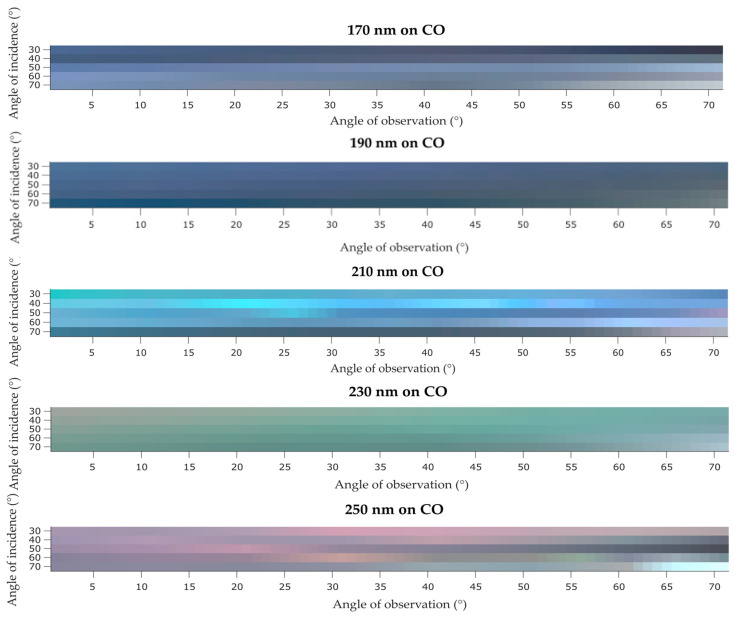
IP-BRDF measurements of CO coated samples.

Since polyester and cotton fabrics did not present iridescence by ocular observation, the highly time-consuming polarized UV/Vis reflectance measurements were not performed for these samples (only 2 samples per day could be measured). In the contrast, the IP-BRDF measurements using the presented methodology, allows around 20 measurements per day. Thus, IP-BRDF was performed for the PES (Figure 15) and CO (Figure 16) samples, to confirm if some iridescence still could be present in these samples. IP-BRDF of PES coated samples showed major alterations in terms of color, as compared to the PA samples, especially for the sample coated with 210 nm PCs, where color changed from green to blue. At high $\alpha_{i}$ and $\alpha_{o}$ a more reddish color can be observed. Also, all PES samples present a lower luminosity parameter L*. The lack of coating uniformity in the PES samples may be the reason for these changes of color and luminosity, as the measured area probably had less photonic crystals, thus the IP-BRDF measurement were affected by a higher percentage of fabric color rather than PC color. This was observed in the samples with 190 and 230 nm PCs. As for samples with 170 and 250 nm PCs, they maintained their color but with darker hues, which is in agreement with the photographs (Figure 4) taken of these samples. Thus, although the self-assembly on PES samples is not as good as in the PA samples, it is possible to affirm that iridescence is present, and being most pronounced in the sample coated with 210 nm PCs.

As for the coated CO samples, all presented a higher luminosity parameter due to the milky aspect on their surfaces as seen in the photographs (Figure 4). Again, when comparing with PA the CO sample with largest color change was the sample coated with 210 nm PCs. In general, the color changed from blue to red at $\alpha_{i}$ 50° and 70° at higher $\alpha_{o}$. The CO sample coated with 230 nm PCs also has a major change when compared to the respective 230 nm PA sample, where the color is mostly green in CO, whereas in PA was mostly red. Overall, the color is changing as $\alpha_{o}$ is increasing for all CO coated samples, confirming an iridescent effect also in these samples. To optimize/improve the structural color on PES and CO samples it will be necessary to do a pre-coating (with biopolymers or conventional finishing agents) of the fabrics in order to smooth their surface [32,33,34]. This will be addressed in future experiments of this research, which are expected to be another step into a bio-ecological and sustainable future.

To further understand the structural color behavior in textiles, IP-BRDF simulations were performed using a different light source. Human color perception varies with different light sources, as their wavelengths are emitted in different regions of the visible spectra. As such, when illuminated with different light sources, the wavelength of the light reflected by the fabric’s surface will change and color perception is different for each illuminant. This is of highest importance to further understand PC color behavior in textiles and, to the authors knowledge, there are no such studies made for textiles. One of the few articles found in the literature report the usage different light source illumination of PCs (He and Lv, [9]), where polystyrene microspheres were 3D-printed onto a black cardboard substrate. The 240 nm PS microspheres presented a green color under a D65 light source, a bluish color under cool white fluorescent (CWF) illuminant, and a yellowish color under illuminant A (IA).

Using IA as light source we made simulations for the PA samples (since they had better results than both the coated PES and CO samples) and compared with the experimental data obtained for PA samples under D50 light source. Our simulations showed that, overall, there were minor differences in terms of color, where the L* parameter was practically the same (ΔL*<1, where $\Delta L*=\left|L_{D50}-L_{IA}\right|$) for most of the PA coated samples under both D50 and IA light sources. The sample coated with 210 nm PCs showed ΔL*=2. A brief comparison of CIElab color coordinates (a* axle − green/red, b* axle − blue/yellow) for both light sources is presented in Table 4.

The PA samples under IA light source illumination are generally greener and yellower, with the exception of the sample coated with 210 nm PCs which is redder and bluer. In terms of the a* axis, the 210 nm coated sample is redder at $\alpha_{i}$ from 30° to 50° and greener at $\alpha_{i}$ above 60°. Samples coated with 230 and 250 nm PCs are in general greener except at high $\alpha_{o}$, where they are slightly redder. In terms of the b* axis, samples coated with 170 and 250 nm PCs are slightly yellower, and sample coated with 190 nm PCs is slightly bluer. These results are in agreement with the study mentioned previously, where color under IA is greener and yellower when compared with color under D50 light source [9]. These color changes are almost imperceptible even by color coordinates analysis, where color differences (Δa and Δb) in most cases are lower than 5 measuring units, in a total range of 256 units for each axle.

## 4. Conclusions

Optical characterization of P(St-MMA-AA) photonic crystals on polyamide, polyester and cotton plain fabrics was performed for five different PC particle sizes. It was determined that the polyamide fabric was the best substrate among the three to mimic nature’s iridescent effect. Polyamide’s tight weave and high surface area, combined with yarns of low Tex, led to a smooth fabric surface with the lowest roughness values among the three fabrics. Consequently, the PA fabric was the only one to present a visible iridescent effect (by ocular inspection) and was further analyzed by angle-dependent UV/Vis reflectance measurements using polarized light. With this analysis it was possible to observe reflection peak shifts and the appearance of new reflection peaks when the angle of incidence was changed. Angle-dependent UV/Vis reflectance and IP-BRDF measurements were proven to be efficient methodologies to characterize iridescence in fabrics, which was also backed up by CIEL*a*b* color coordinates. As for the PES and CO samples, those coated with 210 nm PCs exhibited the most color changes, for both substrates. Overall, PES samples are darker and CO samples have a higher luminosity parameter. This change in luminosity is due to the lack of uniformity in the PC self-assembly, which is a consequence of physical and chemical properties of these fabrics. Simulations with illuminant A suggest that PC color is greener and yellower under this type of light source but maintains the luminosity values, when compared to the experimental data obtained under D50 light source.

The continuous study of photonic crystals in textiles as biomimetics is of major importance, so future generations can have more sustainable methods of coloring textiles. The versatility of photonic crystal textiles in providing functional, sustainable, and aesthetically appealing solutions is driving ongoing research and innovation in biomimicry. Applying PCs to textiles will not only improve the quality and performance of the textiles but also contribute to a more sustainable and technologically advanced future.

## Figures and Tables

**Table 1:** Fabrics characterization according to the ISO standards.

Property Measured	Polyamide	Polyester	Cotton
Thickness (mm)	0.11	0.25	0.30
Yarn linear density (Tex)	3 at weft	8 at weft and warp	20 at weft and warp
	5 at warp		
Number of yarns/cm	45 at weft	32 at weft and warp	30 at weft
	70 at warp		40 at warp

**Table 2:** Surface roughness (Ra) measurements values of uncoated and PC coated fabrics (n = 3).

Fabric	Ra of Uncoated Fabrics (µm)	Ra of PC Coated Fabrics (µm)
Polyamide	6.33 ± 1.99	6.77 ± 1.35
Polyester	16.43 ± 5.51	19.91 ± 3.73
Cotton	17.43 ± 6.27	17.17 ± 5.47

**Table 3:** UV/Vis reflectance peaks of PA coated samples with 170, 210 and 250 nm PCs according to changes in the angle of incident light ($\alpha_{i}$).

$\alpha_{i}$	170 nm PCs on PA UV/Vis Peak (nm)	210 nm PCs on PA UV/Vis Peak (nm)	250 nm PCs on PA UV/Vis Peak (nm)
12°	406	510	641
20°	405	510	639
30°	397	506	639
40°	394	504	636
50°	393	503	633
60°	389	499	627
70°	387	495	622
80°	385	490	619

**Table 4:** Differences in CIEL*a*b* color coordinates of PA coated samples under D50 and IA light sources illuminations. Δa_avg_ and Δb_avg_ are the overall averages of the modular difference for all $\alpha_{i}$ and $\alpha_{o}$ between a* and b* under D50 and IA for each PA coated sample. Δa_avg_ = |a_D50_ − a_IA_|; Δb_avg_ = |b_D50_ − b_IA_|.

Sample	Overall Color Changes under IA	Δa_avg_	Δb_avg_
PA with 170 nm PCs	Greener	5–6 (low $\alpha_{o}$); 1–2 (high $\alpha_{o}$)	<1 (low and high $\alpha_{o}$)
PA with 190 nm PCs	Greener	1–3 (low $\alpha_{o}$); 4–6 (high $\alpha_{o}$)	<1 (low and high $\alpha_{o}$)
PA with 210 nm PCs	Redder and bluer	3–5 (low $\alpha_{o}$); 0–2 (high $\alpha_{o}$)	3–5 (low and high $\alpha_{o}$)
PA with 230 nm PCs	Yellower	<1 (low and high $\alpha_{o}$)	0–2 (low and high $\alpha_{o}$)
PA with 250 nm PCs	Greener	0–2 (low and high $\alpha_{o}$)	~1 (low and high $\alpha_{o}$)

## Data Availability

Data are contained within the article.
